# An improved nonlinear diffusion in Laplacian pyramid domain for cone beam CT denoising during image-guided vascular intervention

**DOI:** 10.1186/s12880-018-0269-1

**Published:** 2018-09-04

**Authors:** Yi Liu, Miguel Castro, Mathieu Lederlin, Adrien Kaladji, Pascal Haigron

**Affiliations:** 1 Shanxi Provincial Key Laboratory for Biomedical Imaging and Big Data, North University of China, Taiyuan, 030051 China; 2 INSERM, U1099, F-35000 Rennes, France; 3 LTSI, Université de Rennes 1, Bât. 22, Campus de Beaulieu, F-35000 Rennes, France; 4 CHU Rennes, Department of Radiology, F-35000 Rennes, France; 5 CHU Rennes, Department of Cardiothoracic and Vascular Surgery, F-35000 Rennes, France

**Keywords:** Nonlinear diffusion, Edge-preserving smoothing, Laplacian pyramid, Low-dose CBCT

## Abstract

**Background:**

Cone-beam computed tomography (CBCT) acquisition during endovascular aneurysm repair is an emergent technology with more and more applications. It may provide 3-D information to achieve guidance of intervention. However, there is growing concern on the overall radiation doses delivered to patients, thus a low dose protocol is called when scanning. But CBCT images with a low dose protocol are degraded, resulting in streak artifacts and decreased contrast-to-noise ratio (CNR). In this paper, a Laplacian pyramid-based nonlinear diffusion is proposed to improve the quality of CBCT images.

**Method:**

We first transform the CBCT image into its pyramid domain, then a modified nonlinear diffusion is performed in each level to remove noise across edges while keeping edges as far as possible. The improved diffusion coefficient is a function of the gradient magnitude image; the threshold in the modified diffusion function is estimated using the median absolute deviation (MAD) estimator; the time step is automatically determined by iterative image changes and the iteration is stopped according to mean absolute error between two adjacent diffusions. Finally, we reconstruct the Laplacian pyramid using the processed pyramid images in each level.

**Result:**

Results from simulation show that the filtered image from the proposed method has the highest peak signal-noise ratio (81.92), the highest correlation coefficient (99.77%) and the lowest mean square error (27.61), compared with the other four methods. In addition, it has highest contrast-to-noise ratio and sharpness in ROIs. Results from real CBCT images show that the proposed method shows better smoothness in homogeneous regions meanwhile keeps bony structures clear.

**Conclusion:**

Simulation and patient studies show that the proposed method has a good tradeoff between noise/artifacts suppression and edge preservation.

**Electronic supplementary material:**

The online version of this article (10.1186/s12880-018-0269-1) contains supplementary material, which is available to authorized users.

## Background

With the progressive development of hybrid operating rooms in vascular surgery, cone-beam CT (CBCT) mounted on a C-arm becomes an increasingly commonly used imaging technology in vascular interventions, such as endovascular aneurysm repair (EVAR), thanks to its capability of 3D imaging of arterial structures and less radiation in comparison with multi-slice CT [1–5]. There are at least two advantages of 3D CBCT acquisition during EVAR. First, detecting any potential complication (endoleaks, stentgraft kinking) that can be treated immediately after the procedure [2–5], i.e., after the deployment of the stentgraft. Secondly, 3D CBCT acquisition (without contrast media injection) was described to fuse the pre-operative CT-scan by means of a 3D/3D rigid bone registration [6–11]. With this fusion imaging technique catheterization and stentgraft deployment can be achieved with a 3D visualization of the vascular tree and leads to a significant decrease of contrast media volume injection.

Although CBCT produces high spatial resolution and offers benefits for patients, there is growing concern on the overall radiation doses delivered to patients due to pre-, intra- and post-operative X-ray imaging during endovascular procedures [12]. As a consequence, it is very important to reduce the radiation dose related to CBCT, not only for patient care, but also for the medical staff to avoid or reduce potential determinist and stochastic risks from radiological procedure [13–15]. However, lowering radiation dose inevitably produces more noise, thus leading to degraded CBCT images with streak artifacts, and decreased contrast-to-noise ratio (CNR) [16]. Noise and streak artifacts suppression is therefore called as a preprocessing step to access a cleaner CBCT image, thus significantly improves the accuracy of subsequent image segmentation and registration.

Generally speaking, there are three categories for reducing noise and artifacts in CBCT: processed before reconstruction [17–19], during reconstruction procedure [20–22] and after reconstruction [23–29]. Since post-processing methods don’t have necessary to access to projection data, various sophisticated filters were applied on the reconstructed images, such as bilateral filtering [23], nonlocal means filtering [24], and nonlinear diffusion filtering [25, 26], most of them consider the strong structural and statistical properties of objects in image space. Recently, dictionary learning and sparse representation were used for reconstruction and enhancement for low-dose X-ray imaging [27–29]. However, they have the limitation of computation time.

The nonlinear diffusion filtering is a useful technique that uses an edge seeking function to encourage diffusion within regions and prohibit it across strong edges. Hence edges can be preserved while removing noise from the image. However, the gradient dependent diffusion, such as the anisotropic diffusion proposed by Perona and Malik [30], cannot effectively distinguish between edges and ramps [31], causing the staircase effect when smoothing ramp regions. Thus, variations of the traditional diffusion filtering (PM) have been proposed to overcome its shortcoming of staircase effect, such as quaternion diffusion [32] and nonlinear complex diffusion [33]. However, most of them worked in a simple scale thus may have the limitation of retaining subtle features. This leads us to consider diffusion in multi-scale.

In this paper, we propose a multi-scale modified nonlinear diffusion (MND) filter using Laplacian pyramid decomposition for CBCT with low radiation dose, with the aim of improving the quality of intra-operative CBCT in EVAR procedures, so that the denoised result is good enough for image interpretation or further processing (e.g. registration with pre-operative CT or post-operative CT). In the modified diffusion filter, the diffusion function is constructed using the gradient magnitude image, rather than gradient values in neighborhood, so that edges can be well preserved and smoothed without introducing obvious staircase effect. And then the proposed Laplacian pyramid-based nonlinear diffusion (LPMND) can reduce noise and streak artifacts while preserving edges and detailed features and completely eliminate the staircase effect.

## Methods

### Laplacian pyramid-based modified nonlinear diffusion

**Fig. 1:**
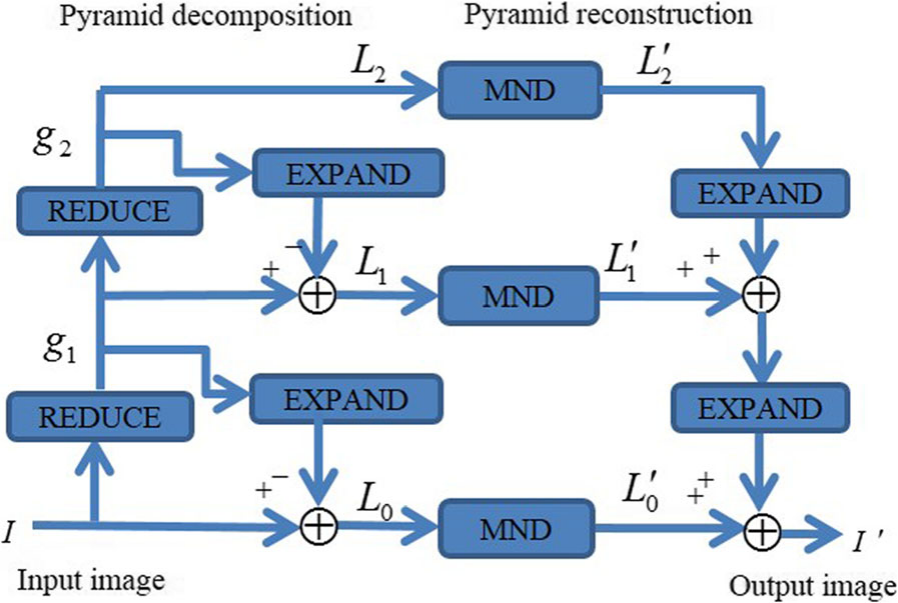
Outline of the proposed LPMND method

The proposed Laplacian pyramid-based modified nonlinear diffusion (LPMND) method is illustrated in Fig. 1. The Laplacian Pyramid is a decomposition of the original image into a hierarchy of images so that each level corresponds to a different band of image frequencies [34]. More details are reported in Additional file 1: Appendix A1. In our method, the degraded image is decomposed into 3 levels with the approximation image as the highest level.Actually noise and useful signal components of an image will be reflected in different levels after decomposition using the Laplacian pyramid. Regard to a degraded image, as noise has high frequency, it mainly exists in the lower pyramid level. However, we need notice that although noise mainly exists in lower level, some also exists in higher level and should be discarded. Similarly, some useful information of structure may exist in the lower level and should be retained. The proposed modified nonlinear diffusion (MND) is therefore applied on the image in each level. Obviously, there are three steps as shown in Fig. 1: (1) transforming the image to be processed into its pyramid domain, (2) restoring all pyramid images by performing MND filter, and (3) reconstructing the Laplacian pyramid using the processed pyramid images in each level.

#### The proposed modified nonlinear diffusion

Although the anisotropic diffusion proposed by Perona and Malik (PM diffusion), more details about it is in Additional file 1: Appendix A2, has been proved to be useful in removing noise (except salt-pepper noise) in homogeneous region, it limits the smoothing at the edge pixels due to permitting more diffusion along the edge than across it and easily generates staircase effect due to the incorrect diffusion at ramp. The reason is that the gradient operator is not a proper measure to detect the ramp features (endpoints) and less effective in noise reduction within the ramp-edge. Several authors suggested using the second derivative to replace the gradient, so that edges and ramps can be effectively distinguished [35, 36]. Gilboa et al. [33] also pointed that the second derivative was a more suitable choice than the gradient because it has a high magnitude near the endpoints and low magnitude elsewhere. That’s, in the diffusion process, the diffusion coefficient should be small near the endpoints and large within the ramp so that noise over the ramp can be reduced and ramp edge can be preserved at the endpoints. However, using the second derivative as the edge indicator may introduce a numerical problem when third order derivatives are computed.

According to the above-mentioned principle, we modified the diffusion function to remove noise while preserving ramp edges. The modified diffusion coefficient *c* is a function of the gradient magnitude image which does not involve the second derivative. It is defined as

1 $$ c\left(g(I)\right)=\frac{1}{1+{\left(g(I)/k\right)}^2} $$

where *I* is the original image and *g*(*I*) is its gradient magnitude image and given by

2 $$ g(I)=\sqrt{{G_x}^2+{G_y}^2} $$

*G*_*x*_ = (*I*(*i*, *j* + 1) − *I*(*i*, *j* − 1))/2, *G*_*y*_ = (*I*(*i* + 1, *j*) − *I*(*i* − 1, *j*))/2.

In this paper, we adopt the explicit numerical scheme, and then the modified nonlinear diffusion (MND) process is

3 $$ {I}_{i,j}^{\left(n+1\right)}={I}_{i,j}^{(n)}+\Delta {t}^{(n)}{\sum}_{Z\in \left\{N,S,W,E\right\}}c\left({g}_Z^{(n)}\right)\kern0.5em {\nabla}_Z{I}_{i,j}^{(n)} $$

Where *Δt* is the time step and ${\nabla}_Z{I}_{i,j}^{(n)}$ means directional derivative. This modified diffusion function not only involves the gradient component of four directions, but also involves far pixels when the corresponding neighboring pixel *g*_*z*_in the gradient magnitude image is computed.

**Fig. 2:**
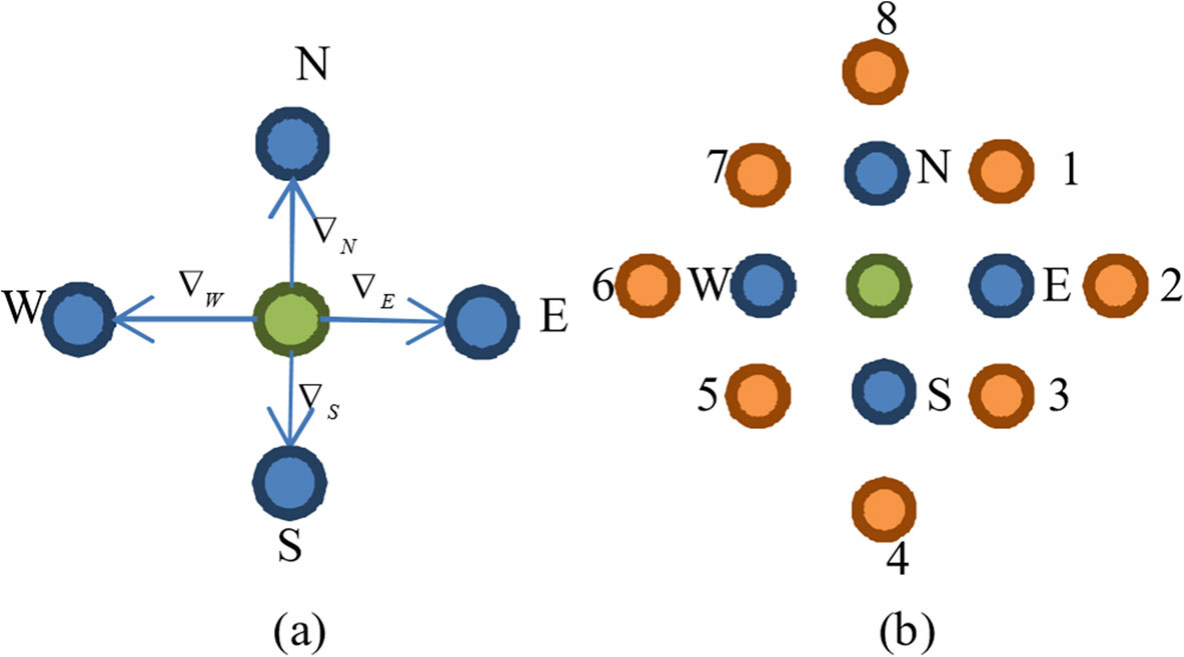
The nonlinear diffusion models. **a** PM model, **b** MND model

Figure 2 illustrates the difference between PM model and MND model. Let the green point be the central pixel to be processed. In PM model, it involves four neighboring pixels (blue points) when calculating the directional diffusion coefficients. In comparison, MND model involves another 8 points (orange points) to calculate the corresponding four neighboring pixels in the gradient magnitude image. The MND model therefore takes more information into consideration when estimating the central pixel. Besides, MND diffusion function only refers to the first derivative, avoiding numerical problems when using the second derivative as the edge indicator. In addition, it is simple to be performed even if it involves more points.

**Fig. 3:**
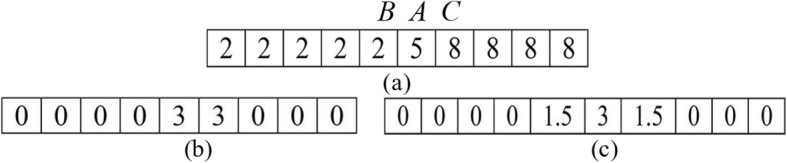
The response of operators to a vertical ramp edge. **a** Ramp edge, **b** response of the gradient operator, **c** response of the gradient magnitude operator

**Fig. 4:**
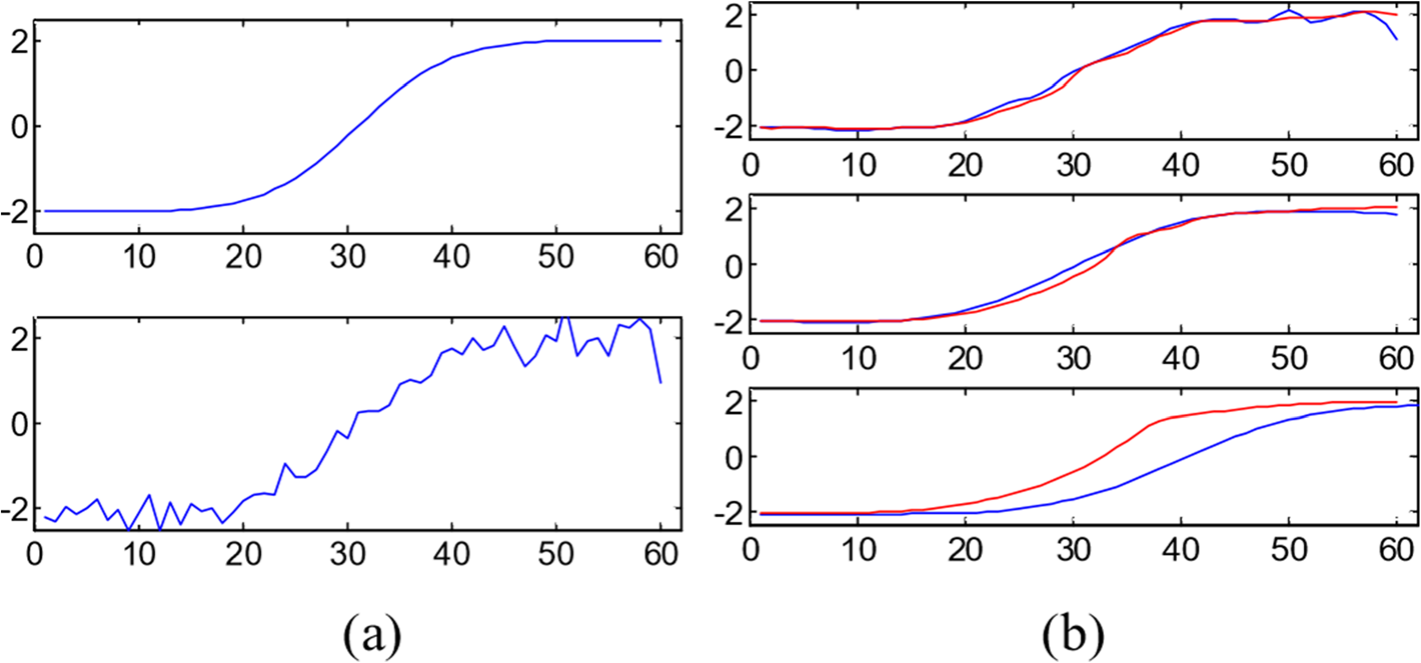
Performs of PM and the MND processes on a ramp edge. **a** Original (top) and noisy ramp signal (bottom), **b** denoised signal from PM process (blue line) and the proposed MND process (red line) with 10, 50, 100 iterations from top to bottom, respectively

Let’s consider the example shown in Fig. 3. Figure 3a shows a sample of ramp edge, Fig. 3b shows the calculated gradients in neighborhood, and Fig. 3c shows the corresponding value in the gradient magnitude version by convolved with *G*_*x*_. With respect to the ramp point marked by *A*, with value 5, the diffusion coefficient in each direction (just West and East in this case) by PM model is *c*_*PM*_ = 1/(1 + (3/*k*)^2^), whereas the corresponding diffusion coefficient in each direction is *c*_*MND*_ = 1/(1 + (1.5/*k*)^2^) by (1). *c*_*MND*_ > *c*_*PM*_, therefore, MND model has a larger flux at the ramp point than PM model as they have the same difference in gray. That’s to say, it has a stronger diffusion to reduce the noise across the ramp. Endpoints of the ramp were marked by *B* and *C*, with value 2 and 8, respectively. For endpoint *B*, the diffusion coefficients in the two models are both *c*^*W*^ = 1 and *c*^*E*^ = 1/(1 + (3/*k*)^2^), thus they have the same flux. The same result can be observed at the endpoint *C*. It is obvious that the two models have the same diffusion strength at endpoints, indicating that MND model has the same capability of preserving endpoints as that in PM model in this example, but has a stronger capability of reducing noise over the ramp. In Fig. 4, we plotted the filtered results of a one-dimensional ramp signal by PM model and MND model. Figure 4a shows the original ramp signal and noisy version contaminated by white Gaussian with SNR = 15.7 dB. Figure 4b shows the denoised results from the PM process and the proposed MND process with 10, 50, and 100 iterations. We observe that in the result from MND process less noise appears with less iterations (10 iterations) and the denoised ramp still keeps its shape with more iterations (100 iterations). This illustrates that ramp edges can be effectively preserved while noise is effectively removed by the MND process in comparison with PM process.

Additionally, Bernardes et al. [37] pointed that using a Gaussian filter for *c* was beneficial to remove speckle noise. We also used a Gaussian filter to smooth the diffusion coefficient because noise in CBCT is complex and may contain speckle noise [38]. Thus, the diffusion coefficient in (1) is redefined as

4 $$ c\left(g(I)\right)={\frac{1}{1+{\left(g(I)/k\right)}^2}}^{\ast }{G}_{\sigma } $$

where ^∗^ is the convolution operator and *G*_*σ*_ is a Gaussian kernel of 3 × 3 size and standard deviation *σ*. When *σ* is small, the filtering of diffusion function in (4) does not change *c* dramatically at edges, whereas it turns diffusion less conservative at speckle points.

#### Parameters determination

Since the selection of threshold *k* is very important in the diffusion process, we prefer an automatic *k* estimated using the median absolute deviation (MAD) estimator proposed by Donoho [38].

5 $$ k=\frac{MAD}{0.6745} $$

where MAD is the median absolute deviation of the wavelet coefficients at the nest level in which most noise exist.

**Fig. 5:**
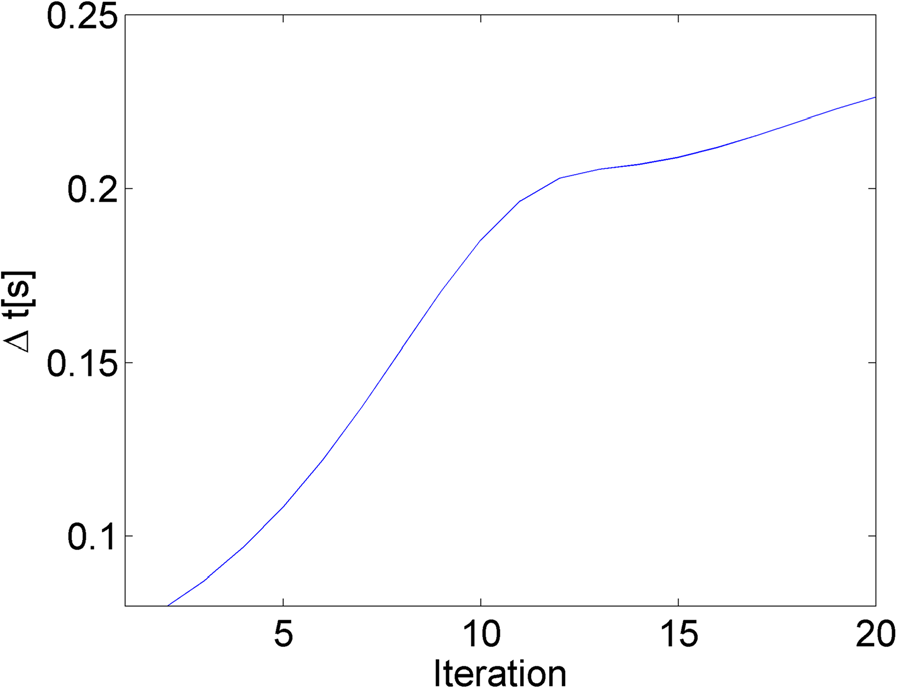
An evolution of the time step using (15)

The time step *Δt* is another important parameter to control the speed of diffusion. It is usually set to a constant that closes to the time step limit of the convergence of the iterative update process. For explicit 2D schemes the maximum time step to achieve stability of the iterative update is 0.25 s. In this work, we adopted an adaptive time step in (6) so that it is small at the initial iterations in which higher values of *c* can be found due to noise and increases until to a steady condition, in which changes of *c* over time are small.

6 $$ \varDelta {t}^{(n)}=\frac{1}{4}\left[a+b\exp \left\{-\left|\mathrm{sum}\left(\raisebox{1ex}{$\partial {I}^{(n)}$}\!\left/ \!\raisebox{-1ex}{$\partial t$}\right.\right)/\mathrm{sum}\left({I}^{(n)}\right)\right|\right\}\right] $$

where $$ \mathrm{sum}\left(\raisebox{1ex}{$\partial {I}^{(n)}$}\!\left/ \!\raisebox{-1ex}{$\partial t$}\right.\right)/\mathrm{sum}\left({I}^{(n)}\right) $$is the fraction of change of the image at iteration *n*, parameters *a* and *b* control the time step with *a* + *b* ≤ 1. We set *a* = 0.25 and *b* = 0.75 in this study. An example evolution of *Δt*^(*n*)^ over the iterative process is shown in Fig. 5.

The stopping criterion is one challenge of diffusion filtering and simply can be stopped manually by setting a fixed number of iterations. However, in the pyramid domain, pyramid images contain different noise levels. Thus, it is difficult to assign a unified value for each level. In this study, we don’t further discuss the stopping criterion, but stop the diffusion using the mean absolute error (MAE) between two adjacent diffusions

7 $$ MAE\left({I}^{(n)}\right)=\sum \limits_{i=1,j=1}^{i=M,j=N}\left|{I}_{i,j}^{(n)}-{I}_{i,j}^{\left(n-1\right)}\right|/\left(M\times N\right) $$

where ${I}_{i,j}^{\left(n-1\right)}$ and ${I}_{i,j}^{(n)}$ are the filtered value at pixel at (*n*-1)*th* and (*n*)*th* iterations, respectively, and *M* and *N* are height and width of the processed image in pyramid domain, respectively. For each level image, the diffusion process stops automatically when the value of MAE is smaller than a preset threshold.

**Fig. 6:**
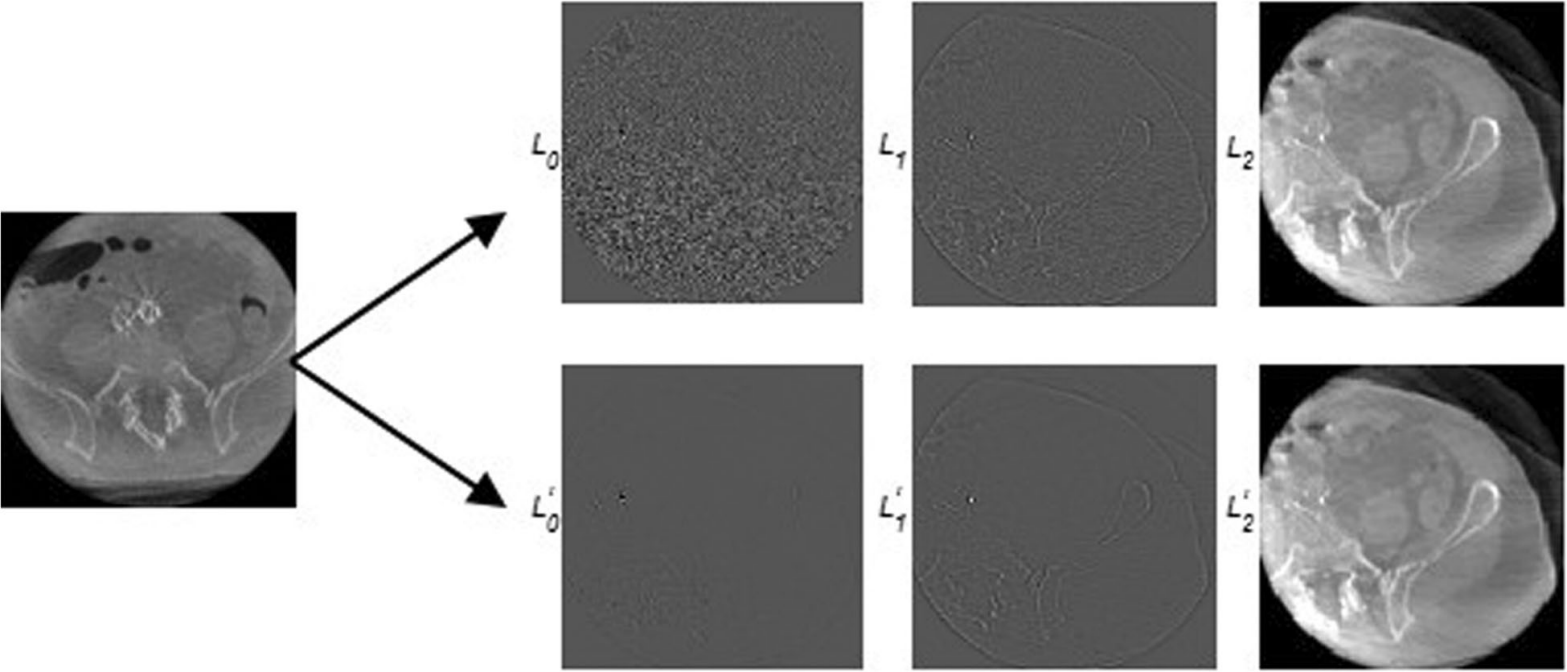
Noise and artifacts suppression in 3 levels of Laplacian pyramid for the degraded CBCT image. Left column shows the original image; right top row shows its 3 levels of Laplacian pyramid; right bottom row shows the restored Laplacian pyramid levels by MND filter

Figure 6 shows one slice of CBCT with poor quality in the left column and its 3 levels of Laplacian pyramid in the right top row. We can observe from the right top row that noise and artifacts play a dominant role in the lowest level and much decrease with the level increases, while progressively coarser features such as edges and structures are prominent in images of higher level. It is obvious that the distributions of noise and artifacts are very different in each level. We therefore should consider different parameters including parameter*k*, time step *Δt*, standard deviation *σ* in (4) and the number of iterations *N* when applying the MND filter on each pyramid level.

The parameter *k* and time step *Δt* could be adaptively determined for each pyramid image still using (5) and (6). With regard to *σ* in (4), it changes in different levels. Therefore, we should do two things: (i) set a high *σ* for the lowest level, since it contains most noise and artifacts, and (ii) reduce *σ* when the level increases and set a small value in the highest level so that it will not change *c* significantly.

The right bottom row in Fig. 6 shows the processed images by the proposed MND filter in each pyramid level. *σ* from level *L*_0_ to *L*_2_was set to 0.8, 0.5, and 0.1, respectively. We can see that the MND filter leads to artifacts suppression effectively in the lower pyramid levels, particularly in the lowest level, yet modifies little in the coarsest level.

### CBCT simulation and acquisition

**Fig. 7:**
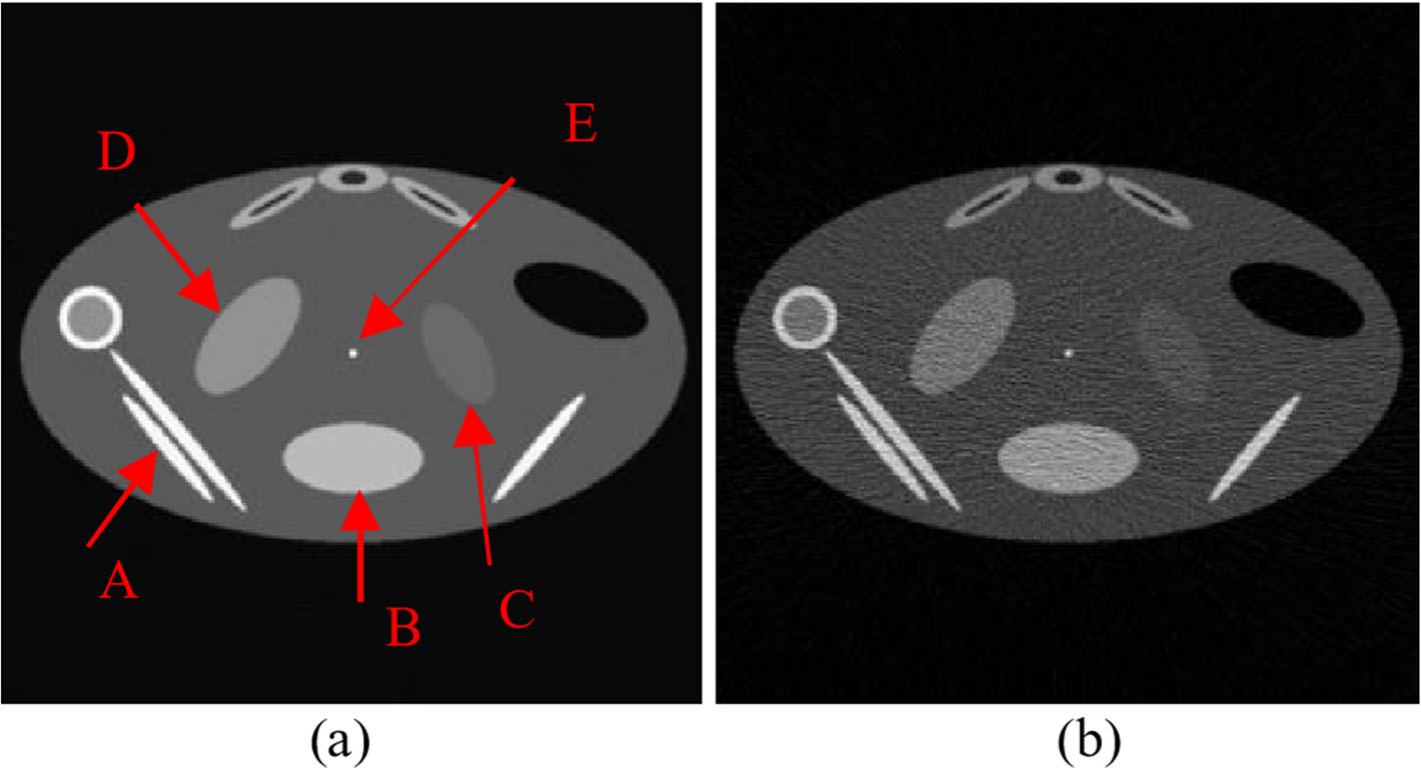
CBCT images reconstructed by FDK for digital phantom. **a** Clean CBCT image (as the reference image), **b** noisy CBCT image

In order to evaluate the performance of the proposed LPMND, we performed experiments on simulated and real abdominal CBCT images. The simulation experiment was conducted by using a numerical phantom (shown in Fig. 7a) which imitates the structures of a real abdominal CBCT image, and thus we can evaluate the proposed method quantitatively. A large ellipse object in the center of the phantom was used as reference “background tissue”. Object A was used to mimic bony structures and elliptical objects B, C, D were used to mimic soft tissues. Object E in the center simulates the guidewire inserted into the artery and was also chosen for a spatial resolution test. This phantom was simulated for the central slice in the CBCT imaging.

This phantom was then back-projected according to the following scanning: The distance of X-ray source to the center of rotation was 541 and 984 angular samples evenly spanned on a circular orbit of 360°. The central slice was then reconstructed from this noise-free sinogram using the well-known FDK algorithm [39], as shown in Fig. 7a, and can be as the reference image in the performance analysis. To simulate a FDK reconstructed CBCT image acquired by a low-dose protocol, we generated a noisy sinogram by adding signal-dependent Gaussian noise to the noise-free sinogram, according to the noise model in (8). This noise model was presented by Li et al.*..* [40] and Wang et al [41] with the content that the projection data after system calibration and logarithm transformation was approximately Gaussian distributed with a non-linear dependency between sample mean and variance as follows.

8 $$ {\sigma^2}_{p_s}=f\exp \left({\overline{p}}_s/\eta \right) $$

where ${\overline{p}}_s$ and ${\sigma}_{p_s}^2$ is the mean and variance at detector bin *s*. *η* and *f* are parameters determined by system settings. In our study, *η* and *f* were set to 22,000 and 200, respectively. The CBCT image was then reconstructed by FDK, shown in Fig. 7(b). Note that the simulated image contains obvious noise and streak artifacts that is the characteristic of low-dose CBCT.

For experiments using patient data, the study was approved by the ethics committee of Rennes University Hospital (France) on April 10, 2016. Patient informed consent was obtained for being registered anonymously in the database. We used a Siemens Artis zeego C-arm system in the operation room to obtain a CBCT for a patient during an EVAR. Four protocols are preset in this system: they are 5sDR protocol, 5sDSA protocol, 8sDR protocol and 20sDR protocol. Low-dose intra-operative CBCT was realized using a 5sDR protocol. The 5sDR protocol has 5-s acquisition time capturing 133 frames at 30 frames/second (f/s). The 5sDSA protocol is used for acquiring a digital subtraction angiography (DSA). The 5sDSA protocol has the same characteristics of the 5sDR but it realized two rotations, the first one is used to acquire the mask, and the second one is synchronized with the injection with the injection of contrast medium. Other acquisition parameters were: the distance from the X-ray source to detector is 1199 mm, the field of view (FOV) is 100 mm × 100 mm, the slice thickness is 0.4804 mm, the size of one slice of the CBCT is 512 × 512 and pixel spacing is 0.4804 mm × 0.4804 mm. The related scanning parameters were 91KV, 243mAs.

### Performance evaluation

To evaluate the performance of noise reduction, we computed several well-known image performance metrics: mean square error (MSE), peak signal-to-noise (PSNR), and correlation coefficient (CC). MSE and the PSNR are error metrics used to test the quality of filtered image. MSE represents the cumulative squared error between the filtered and the original image, while PSNR represents a measure of the peak error. CC indicates the degree of spatial similarity between the original and the filtered image. Definitions of these metrics are in (9)–(11).

9 $$ MSE=\sum \limits_{i=1,j=1}^{i=M,j=N}{\left({\widehat{I}}_{i,j}-{I}_{i,j}\right)}^2/\left(M\times N\right) $$

10 $$ PSNR=20{\log}_{10}\left(\frac{2^B-1}{\sqrt{MSE}}\right) $$

11 $$ CC=\sum \limits_{i=1,j=1}^{i=M,j=N}\left({\widehat{I}}_{i,j}-{\mu}_{\widehat{I}}\right)\left({I}_{i,j}-{\mu}_I\right)/\sqrt{\sum \limits_{i=1,j=1}^{i=M,j=N}{\left({\widehat{I}}_{i,j}-{\mu}_{\widehat{I}}\right)}^2{\left({I}_{i,j}-{\mu}_I\right)}^2} $$

where *I* and *μ*_*I*_ are the original image and its mean value, respectively. $\widehat{I}$ and ${\mu}_{\widehat{I}}$ are the filtered image and its mean value, respectively. *B* represents the bits per sample. A good result should have small MSE, high PSNR and CC.

Besides, to quantify localized differences, we evaluated the contrast-to-noise ratio (CNR) over specific regions of interest (ROIs). The CNR measures the contrast between a ROI and the background region. It is generally defined as

12 $$ CNR=\left|{\mu}_R-{\mu}_B\right|/\sqrt{\sigma_R^2+{\sigma}_B^2} $$

where *μ*_*R*_ and *σ*_*R*_ are the mean and standard deviation of the ROI, including the homogeneous region and *μ*_*B*_ and *σ*_*B*_ are the mean and standard deviation of the background region, respectively. To evaluate the performance of resolution preservation, we also analyzed the spatial resolution by using the modulation transfer function (MTF), which was approximately obtained by using the object E in Fig. 7(a). A ROI placed at the center of the object was extracted and the line spread function (LSF) then was obtained by integrating the ROI in *y*-direction, finally MTF can be obtained by calculating the Fourier transform of the normalized and zero-padded LSF. In addition, the sharpness measurement tool proposed by Taubmann et al. [42] was used to support comparison of edge sharpness in images. This tool not only gives a plot of sharpness estimates along an edge for images to be tested, but also computed sharpness values.

## Results

In this section, we evaluate the proposed LPMND filter by using both simulated image and real abdominal CBCT data. The simulation study gives quantitative performance analysis and the patient study demonstrates the practical applicability of the proposed filter in the procedure of EVARs. In each study, we compared the LPMND filter with other filters including PM filter with diffusion function in (1), nonlinear complex diffusion filter (NCDF), bilateral filter (BF) and nonlocal means filter (NLMF). For LPMND, *α*in Gaussian kernel for REDUCE and EXPAND operators was set to 0.375 and the images were decomposed into three pyramid levels as more levels didn’t guarantee improved performances. “db2” wavelet was used to calculate the parameter *k* in (5). The standard deviation *σ* of Gaussian filter in (4) was set to 0.8, 0.5, and 0.1 from the lowest level to the highest level, respectively. These parameters were hand-tuned set to obtain the possible optimization based on trial errors.

### Results from simulated image

**Fig. 8:**
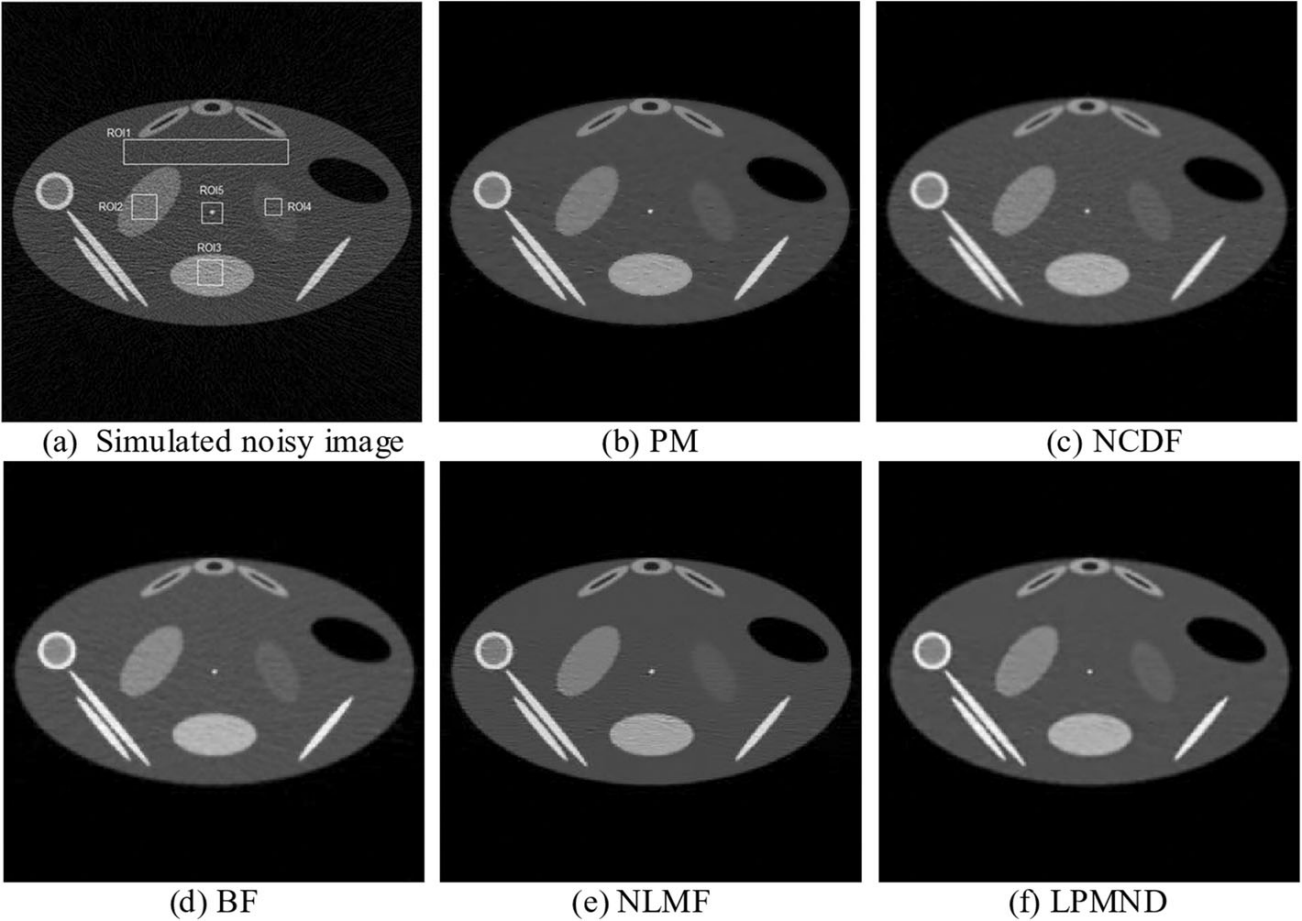
Comparison of the simulated image. **a** Simulated low-dose CBCT image, **b**-**f** images filtered by PM, NCDF, BF, NLMF, and LPMND, respectively

The five different filters were applied on the simulated image in Fig. 7b, in which plenty of noise and artifacts exist. The time step was 0.15 and 0.055 for PM and NCDF, respectively. The parameter *k* of PM and NCDF was also determined by (5). LPMND, PM and NCDF followed the same stop criterion, i.e., MAE criterion in (7), and the thresholds for the MAE in PM, NCDF and LPMND were all set to 0.11. The search window and similarity window in NLMF ware set to 21 × 21 and 7 × 7, and the filtering parameter was 18, whereas in the BF, the standard deviations of closeness function and similarity function were 3 and 100, respectively. All of the parameters in each filter were appropriately set to achieve a matched noise level. PSNR values in the filtered images from PM, NCDF, BF, NLMF, and LPMND were 79.98, 78.89, 80.37, 80.97, and 81.92, respectively. Figure 8 shows the comparison of different filters. PM doesn’t give a satisfactory result due to the fact that obvious staircase effect exists in the filtered image, although it has a good capability of preserving edge. NCDF behaves well on avoiding staircase effect but is less satisfactory on suppressing enough noise and streak artifacts. Like NCDF, BF don’t perform well on suppressing noise and streak artifacts, while NLMF generates wavy artifacts in the filtered image, as shown in Fig. 8e. In comparison, the proposed LPMND gives a better tradeoff between noise/artifacts reduction and edge preservation. From Fig. 8f, we can observe that weak edges are well enhanced.

To quantitatively compare the performances of different filters, we then calculated the quality parameters MSE and CC at the matched PSNR, as shown in Table 1. Furthermore, we calculated the CNR and ENR to quantify the local performance in regions of interest. ROI1 indicated by a rectangle in Fig. 8a was seen as the background region, and three homogeneous regions (ROI2, ROI3, and ROI4) were used to calculate CNR and ENR values. Table 2 lists the CNR value for each region, respectively. It is obvious from Table 1 that LPMND has the lowest value of MSE and highest values of CC in comparison with the other filters, meaning it is most closed to the original image. Furthermore, it has highest values of CNR for each homogeneous region, as shown in Table 2.

**Table 1:** MSE, PSNR, and CORR values of different filtered images

	Noisy	PM	NCDF	BF	NLMF	LPMND
PSNR	69.83	79.98	78.89	80.37	80.97	81.92
MSE	446.88	43.17	55.43	39.41	34.38	27.61
CC(%)	96.40	99.63	99.53	99.67	99.71	99.77

**Table 2:** CNR values of three ROIs

	Noisy	PM	NCDF	BF	NLMF	LPMND
ROI2	2.15	11.01	8.24	10.27	11.54	13.30
ROI3	3.36	11.46	11.70	16.20	10.24	19.41
ROI4	0.62	3.25	2.45	3.21	3.75	4.80

**Fig. 9:**
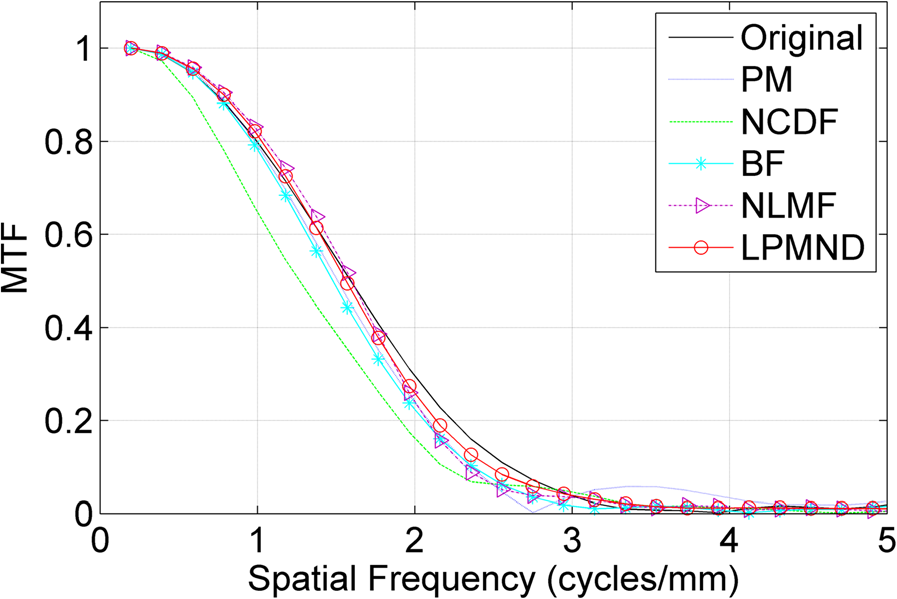
Comparison of MTF curves from the original image and the filtered images in Fig. 8b-f

**Fig. 10:**
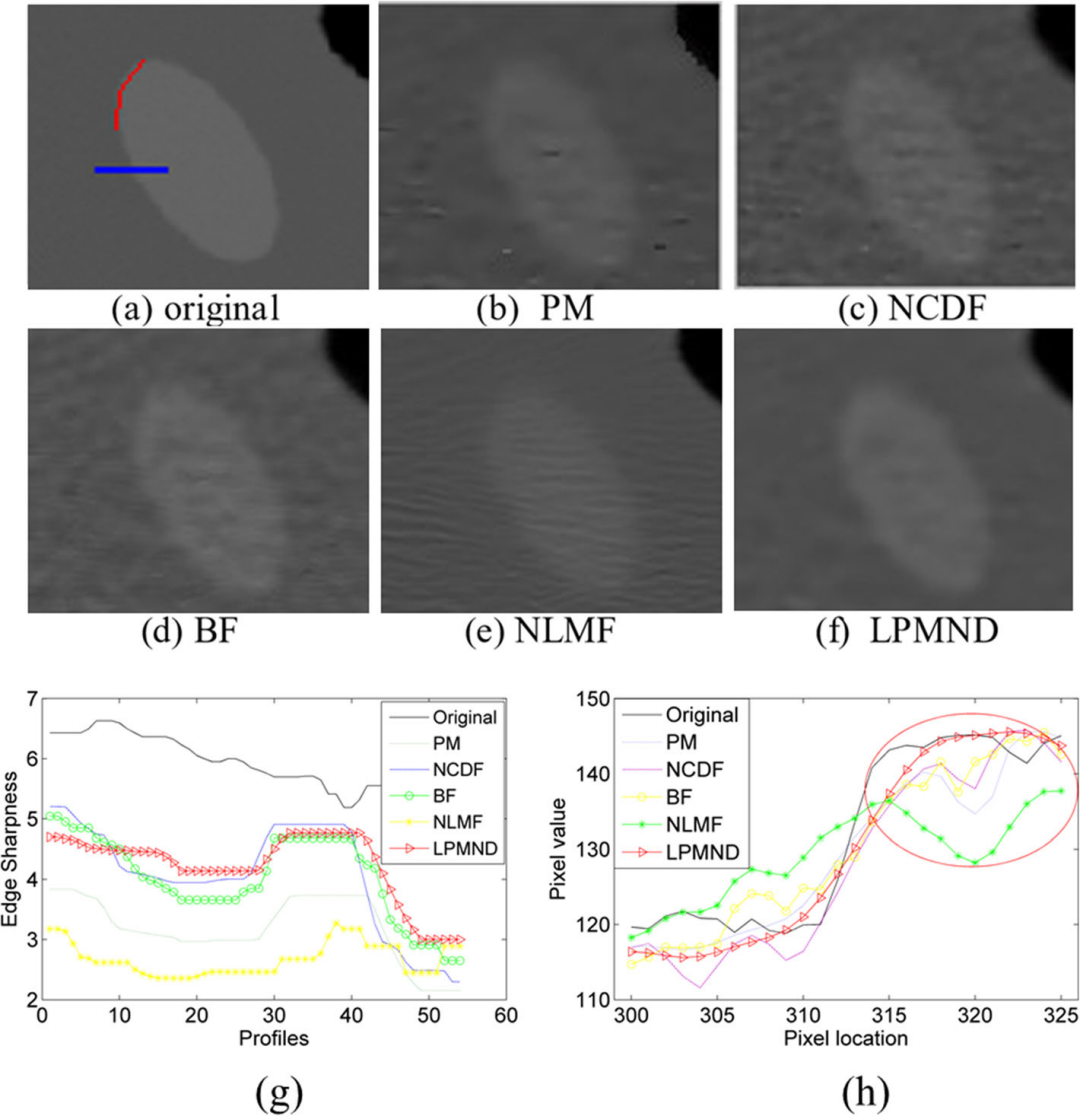
Comparison of sharpness for a low-contrast edge. **a**-**f** Enlarged ROI4 of the original image and the filtered image by PM, NCDF, BF, NLMF, and LPMND, respectively. **g** The plot shows the corresponding sharpness estimates along the edge (indicated by red curve) for different filtered image. **h** Comparison of horizontal profiles, indicated by the blue line in Fig. 10a

To measure the resolution of the filtered images, ROI5 indicated in Fig. 8a was used to calculate the MTF. Figure 9 depicts the MTF curves of the original image and the filtered images in Fig. 8b-f. We observe that the MTF curve of LPMND filtered image is most closed to that of the original image, compared with results from PM, NCDF, BF, and NLMF. It means that the spatial resolution in the LPMND filtered result is higher than those filtered by the other four filters. In order to evaluate the performances of edge preservation, we showed an enlarged low-contrast region (ROI4) and analyzed its edge by using the sharpness measurement tool. The corresponding enlarged images related to Fig. 8b-f are shown in Fig. 10b-f with the enlarged one of original image shown in Fig. 10a. The tested edge is indicated by the red curve in Fig. 10a. Figure 10g plots the sharpness estimates along the edge for different filtered image. The computed edge sharpness estimates are summarized in Table 3. In addition, Fig. 10h shows the profiles passing ROI4 indicated by a blue line in Fig. 10a. Through those profiles, it can be observed that NLMF failed on preserving edges of the low-contrast region; PM, BF and NCDF don’t have good behaviors of smoothing the homogeneous region, seeing the part marked by a red circle; By contrast, LPMND likely reaches a compromise between smoothing and edge preserving.

**Table 3:** Edge sharpness of low-contrast edge in different filtered images

	Original	PM	NCDF	BF	NLMF	LPMND
Sharpness	5.999	3.136	4.079	4.107	2.508	4.441

### Results from real CBCT images

**Fig. 11:**
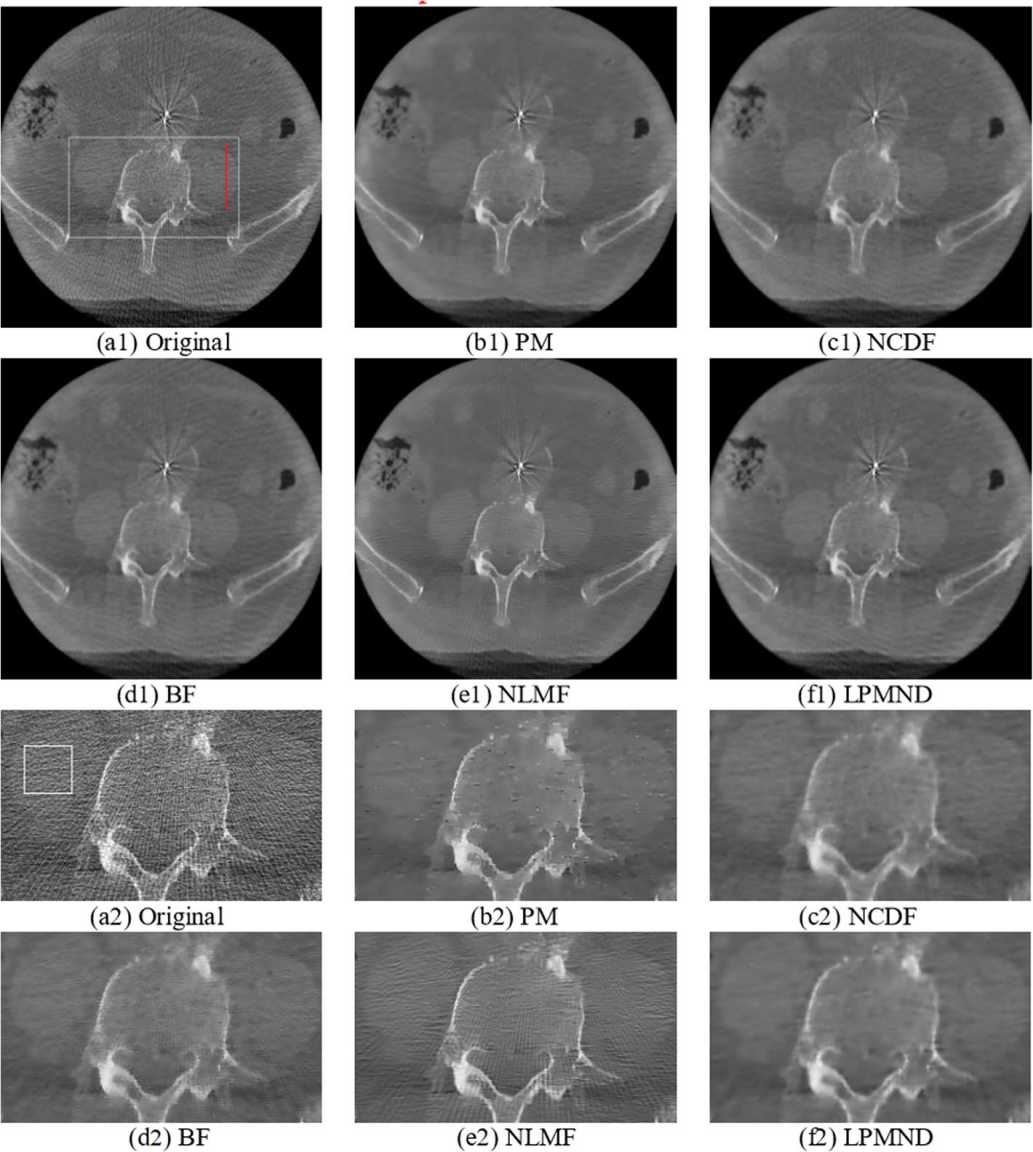
One slice of the CBCT data from one patient and its enlarged region which is indicatd by a white square in (**a1**). **a1** the original image, **b1**-**f1** filtered image by PM, NCDF, BF, NLMF and LPMND, respectively. **a2**-**f2** Corresponding enlargements in (**a1**-**f1**). Display window: [− 1024 HU, 1976 HU]

**Fig. 12:**
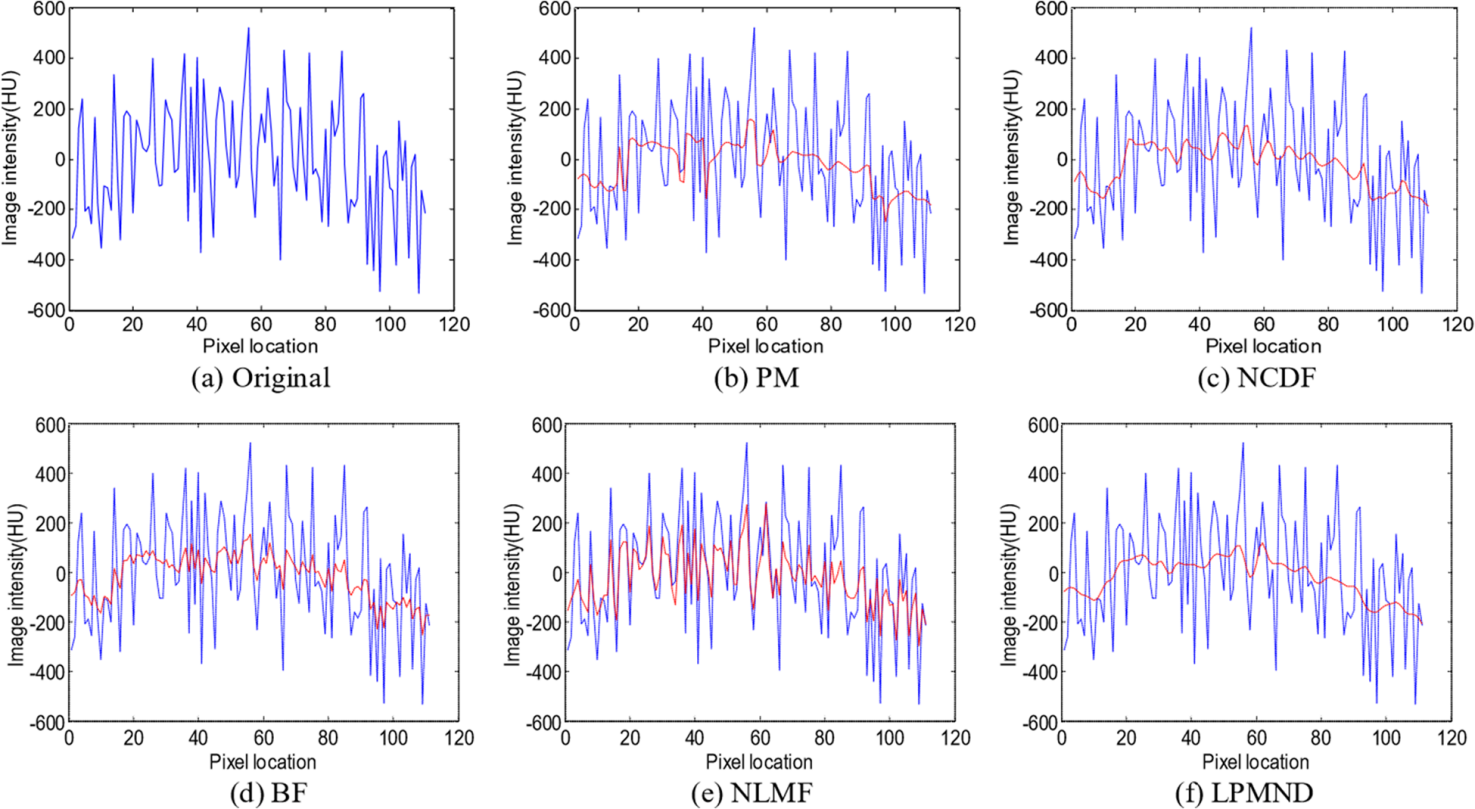
Comparison of vertical profiles, indicated by the red line in Fig. 11 (a1), for different filtered images as shown in Fig. 11 (b1-f1). (**a**) Original image, (**b**)-(**f**) images filtered by PM, NCDF, BF, NLMF, and LPMND, respectively

In this section, we used the intra-operative CBCT data of an AAA (abdominal aortic aneurysm) patient to evaluate the effectiveness of the proposed filter. The original image of slice 152 is shown in Fig. 11(a1) and the filtered results obtained by PM, NCDF, BF, NLMF and LPMND are shown in Fig. 11(b1-f1), respectively. The thresholds for the MAE in PM, NCDF and LPMND were all set to 2. The time step was 0.24 and 0.1 for PM and NCDF, respectively. The search window and similarity window in NLMF were set to 7 × 7 and 3 × 3, and the filtering parameter was 150, whereas in the BF, the standard deviations of closeness function and similarity function were 3 and 500, respectively. All of parameters in each filter were hand-tuned set to obtain possible best results based on trial errors. For a better illustration, the enlarged regions of bony structure and renal structure, marked by a white block in Fig. 11 (a1), are illustrated in Fig. 11(a2-f2). We can see that the filtered images obtained by PM, BF and NLMF have sharp edges, but PM yields staircase effect while BF and NLMF (in particular) are less helpful for artifacts suppression. NCDF behaves well on smoothing homogeneous region, but makes the bony structures blurred. In comparison, LPMND outperforms other filters on suppressing artifacts and preserving edges. Furthermore, a vertical profile (indicated by a red line in Fig. 11(a1) is plotted in Fig. 12 for the original image and each filtered result. The filtered profiles are overlapped by the original profile for comparison. Through these profiles, we can observe that LPMND filtered image shows better smoothness in homogeneous regions.

**Fig. 13:**
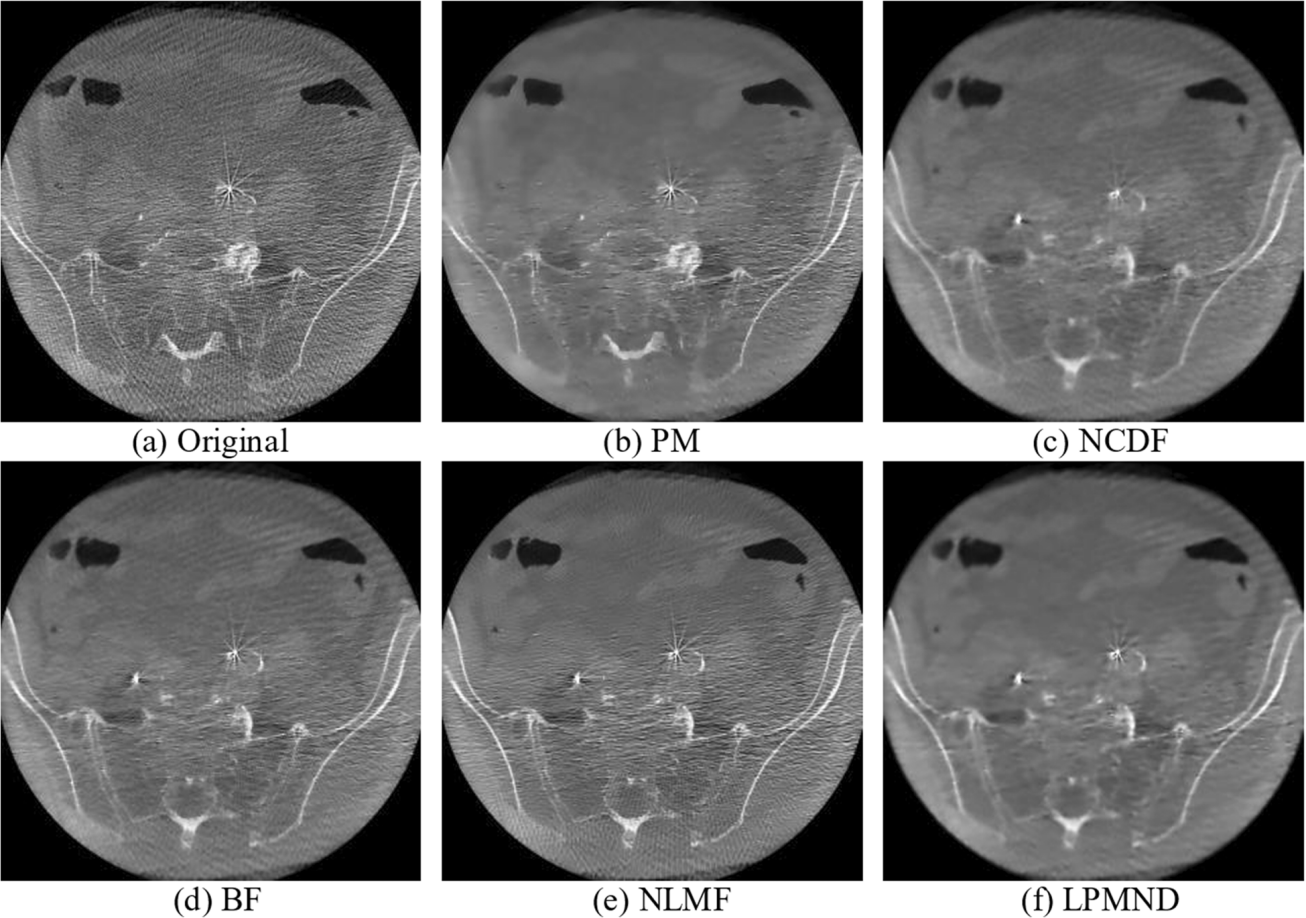
Comparison of slice 29. Display window: [− 1024 HU, 1976 HU]. (**a**) Original image, (**b**)-(**f**) images filtered by PM, NCDF, BF, NLMF, and LPMND, respectively

In practice, the noise level changes across slices even in the same CBCT data, we therefore selected another slice (slice 29) that contains more artifacts to test the proposed filter. Figure 13 shows the comparison of processed images by different filters. In order to evaluate the consistent performance of different filters, the parameters in each filter were kept the same as those used for slice 152. It can be observed that PM, NLMF and BF cannot suppress artifacts effectively compared with NCDF and LPMND. On the other hand, the bony structures in the NCDF filtered image are a little blurred, whereas the LPMND filtered image has clear bony structures. Thus, the LPMND filter may have superiority for practical applications.

We also calculated the average time for one CBCT data of 365 slices under the environment that OS: 64-bit; Windows 7; CPU: Intel Core(TM) i7, 8G RAM. The computation time for PM, NCDF, BF, NLMF and the proposed LPMND is about 6 min, 43 min, 19 min, 59 min, and 4 min, respectively.

## Discussion

The number of endovascular aneurysm repair is yearly growing and concerns about radiation are more topical than ever. Works dealing with low-dose X-ray imaging protocols are appropriate for optimization of the clinical use of this acquisition. Therefore, noise and artifacts caused by low dose protocols in intra-operative CBCT acquisition need to be addressed. Our study aimed to improve the quality of intra-operative CBCT acquired with low dose protocols so that a more accurate segmentation or registration can be carried out, thus to guide and monitor the insertion procedure. Furthermore a post-procedural CBCT with good noise reduction could better help assess early complications compared to classical 2D angiography. In practice, we can restore the projection image or the reconstructed images, or obtain the final reconstructed image using a statistical image reconstruction (SIR) algorithm. Projection denoising takes noise properties in projections into account, yet has the potential disadvantage that the definition of edge in projection data is not definite, resulting in sharpness loss in image domain. SIR methods more focus on reconstruction rather than denoising techniques, utilizing the noise properties in projections and image prior information. While more sophisticated physical modeling and image reconstruction can be viewed as a superior approach, they always have high computation load and are highly dependent on special scanner model, i.e., requiring more detailed information such as scanning geometry, correction physics, etc. This limitation appeals a more broadly used denoising method that can perform on different systems, and leads us to think more about denoising after reconstruction. Image post-processing techniques, working in the image space alone, are retrospectively applied and relatively simple to implement. For the reasons mentioned above, we choose to implement noise and artifacts suppression in image space.

Laplacian pyramid is a very useful tool for multi-scale analysis. Unlike sub-band decomposition in wavelet transform, REDUCE and EXPAND operators generate a series of low-pass and band-pass images that contain approximation and detailed images without requiring quadrature mirror filters. The pyramid offers a useful image representation for denoising tasks, since it can represent information that is localized in both space and spatial frequency in each level [43]. Since CBCT noise in image space is difficult to model accurately and has strong correlations, the Laplacian pyramid is considered to remove much of the pixel-to-pixel correlation.

In this study, we have proposed a modified nonlinear diffusion filter in Laplacian pyramid to reduce noise and artifacts without damaging image features. It consists of the following three steps: (1) Laplacian pyramid decomposition, (2) performance of the modified nonlinear diffusion filter in each pyramid level, and (3) reconstruction from the processed pyramid images. We modified the diffusion function of PM model so that it can remove noise across ramp edges, to a large extent avoiding transforming ramp edges in to stairs. The staircase effect is then remedied by multi-scale decomposition through Laplacian pyramid. There are several advantages of this proposed method beyond that. First, the threshold parameter *k* in diffusion function is adaptive and automatically determined by (5). Secondly, we adopted an adaptive time step which changes according to iterative image changes. And then the speed of diffusion process is accelerated as well. Thirdly, the diffusion is automatically stopped using the MAE between two adjacent diffusions, rather than manually stopped by setting a fixed number of iterations. Furthermore, the value of MAE for different level image in pyramid domain could be fixed the same according to our experiments, avoiding multi-choice in multi-scale decomposition. In addition, in view of the computation cost, the proposed method is fast and easy to implement, thus is possible to be used for clinical applications.

In this work, our effort was focused on the noise and streak artifacts suppression for the intra-operative CBCT during an EVAR. We have performed simulated and real CBCT images to evaluate the proposed method. Noise and artifacts lead to a degraded image, especially in low-contrast regions. The proposed method can suppress noise and streak artifacts meanwhile protect low-contrast regions. We also compared the proposed method with other edge-preserving filters through quantitative analysis in simulation study and qualitative analysis in patient study. The MTF study shows that LPMND can produce good image resolution (Fig. 9), and the sharpness estimation shows that it works well on preserving edges of low-contrast regions (Fig. 10 and Table 3). Actually, we have verified that the proposed method performed well on clinical CBCT data with full slices. This method could be used to improve results of further processing (e.g. CT – CBCT registration).

There are also some drawbacks of this proposed method. One is the selection of parameter *k.* Actually, *k* plays an important role in diffusion procedures and should correspond to the noise level. However, the noise level in CBCT is very complex, resulting in the difficulty to establish a noise model. The performance of the proposed filter may be further improved with an accurate noise model to determine *k*. Furthermore, although the proposed method performs well for the streak artifacts caused by noise, it is not very effective for the metal artifacts caused by high-attenuation objects, e.g., the inserted guidewire. In fact, metal artifacts remove is another challenge for CBCT imaging. Finding an effective solution to suppress metal artifacts is necessary and is considered in our next step. In addition, the proposed method is presented on 2D case, making the information from the successive slices not to be concerned. Although some other methods were proposed in three dimension, such as the KL-PWLS method in [20], the computational load is an issue to be resolved. We therefore processed the CBCT data slice by slice, and in fact the proposed method was satisfactory when we used it in practical applications.

## Conclusion

We have presented a Laplacian pyramid-based nonlinear diffusion filter for intra-operative CBCT in endovascular aneurysm repair. The multi-scale diffusion can effectively remove noise and artifacts, improve the CNR for low-contrast regions, and meanwhile preserve edges and detailed features. Compared with some other edge-preserving filters, the proposed method has shown better performance for the CBCT in EVAR procedures.

## Additional file

Additional file 1: Appendix. The Laplacian Pyramid (A1), Anisotropic Nonlinear Diffusion (A2). (PDF 62 kb)

## Abbreviations

AAA: Abdominal aortic aneurysm
BF: Bilateral filter
CBCT: Cone-beam computed tomography
CC: Correlation coefficient
CNR: Contrast-to-noise ratio
DSA: Digital subtraction angiography
EVAR: Endovascular aneurysm repair
FOV: Field of view
LPMND: Laplacian pyramid-based modified nonlinear diffusion
LSF: Line spread function
MAD: Median absolute deviation
MND: Modified nonlinear diffusion
MSE: Mean square error
MTF: Modulation transfer function
NCDF: Nonlinear complex diffusion filter
NLMF: Nonlocal means filter
PSNR: Peak signal-to-noise ratio
ROI: Region of interest
SIR: Statistical image reconstruction
