# Supplementary material for: An improved nonlinear diffusion in Laplacian pyramid domain for cone beam CT denoising during image-guided vascular intervention
Source: BMC Med Imaging. 2018 Sep 4;18:25. doi: 10.1186/s12880-018-0269-1 (PMC6122689; doi:10.1186/s12880-018-0269-1)
Supplement: Supplementary file 1 — Appendix. The Laplacian Pyramid (A1), Anisotropic Nonlinear Diffusion (A2). (PDF 62 kb) [file 12880_2018_269_MOESM1_ESM.pdf]

Appendix

A1. The Laplacian Pyramid

The Laplacian Pyramid decomposition is done by taking the difference of levels in the Gaussian pyramid. The Gaussian pyramid is a multi-resolution image representation through the operation REDUCE, which is carried out by convolving the image with a Gaussian kernel and down-sampling it by a factor of two in each dimension. When this operation is used multiple times, it generates a sequence of low-pass filtered images. The Laplacian pyramid is a series of band-pass filtered images with each level built by using the EXPAND operator, which up-samples the low-pass image by a factor of two (inserting zero after each pixel) and then convolves it with the same Gaussian kernel. Thus the Laplacian pyramid level can be obtained by subtracting each Gaussian pyramid level from the next lower level in the pyramid.

The Gaussian kernel is designed to make the center pixel gets more weight than the neighboring ones and their sum is 1. The Gaussian kernel is given by

$$\omega(r,c)=\hat{\omega}(r)\,\hat{\omega}(c),\ \hat{\omega}(r)=[\frac{1}{4}-\frac{\alpha}{2},\frac{1}{4},\alpha,\frac{1}{4},\frac{1}{4}-\frac{\alpha}{2}] \quad (1)$$

where α is usually in the range [0.3,0.6].

Let I be the original image, the low-pass filtered image at level l ($0 \le l \le s$) is defined as

$$\begin{bmatrix} g_0(x,y)=I \\ g_l = REDUCE(g_{l-1}) \end{bmatrix} \quad (2)$$

Mathematically, the level g_l of the Gaussian pyramid is obtained as follows

$$g_l = REDUCE(g_{l-1}) = \sum_m \sum_n \omega(m,n) g_{l-1}(2x+m, 2y+n) \quad (3)$$

The Laplacian pyramid is a series of band-pass filtered images with each level built by using the EXPAND operator, which up-samples the low-pass image by a factor of two (inserting zero after each pixel) and then convolves it with the same Gaussian kernel. It acts as the reverse of REDUCE

$$EXPAND(g_l) = 4\sum_m \sum_n \omega(m,n) g_l\left(\frac{x+m}{2},\frac{y+n}{2}\right) \quad (4)$$

Thus the Laplacian pyramid level L_0, L_1, L, L_s can be obtained by subtracting each Gaussian pyramid level from the next lower level in the pyramid.

$$\begin{aligned} L_l &= g_l - EXPAND(g_{l+1}) \\ &= g_l - EXPAND(REDUCE(g_l)) \end{aligned} \tag{5}$$

As there is no image g_{s+1}, we say $L_s = g_s$. It can be shown that the original image g_0 can be reconstructed from its pyramid representation by simply reversing the decomposition steps.

A2. Anisotropic Nonlinear Diffusion

The anisotropic diffusion, one of the famous nonlinear diffusions, was first proposed by Perona and Malik [30]. The main idea of anisotropic diffusion filter is that image features, such as the discrete gradient, can be used to derive different intensities of diffusion in different directions. The equation for diffusion process described by is

$$\frac{\partial I}{\partial t} = \nabla \cdot \left(c\left(\nabla I \right) \nabla I \right), \quad I_{t=0} = I_0 \tag{6}$$

where $\nabla \cdot$ is the divergence operator, and ∇ is the gradient operator. c is the diffusion coefficient which is a nonnegative monotonically decreasing function with $c\left(0\right)=1$ and is used to control the rate of diffusion along different directions. I_0 is the original image. The numerical implementation is given by

$$\begin{aligned} I_{i,j}^{n+1} = I_{i,j}^{n} + \Delta t \cdot \Big(& c\left(\nabla_N I_{i,j}^n\right) \cdot \nabla_N I_{i,j}^n + c\left(\nabla_W I_{i,j}^n\right) \\ & \cdot \nabla_W I_{i,j}^n + c\left(\nabla_S I_{i,j}^n\right) \cdot \nabla_S I_{i,j}^n + c\left(\nabla_E I_{i,j}^n\right) \cdot \nabla_E I_{i,j}^n \Big) \end{aligned} \tag{7}$$

where Δt is the time step, and the dirctional derivative estimates are: $\nabla_N I_{i,j}^n = I_{i-1,j}^n - I_{i,j}^n$, $\nabla_S I_{i,j}^n = I_{i+1,j}^n - I_{i,j}^n$, $\nabla_W I_{i,j}^n = I_{i,j-1}^n - I_{i,j}^n$, $\nabla_E I_{i,j}^n = I_{i,j+1}^n - I_{i,j}^n$. The diffusion function suggested by Perona and Malik is

$$c\left(x\right) = \frac{1}{1+\left(x/k\right)^2} \tag{8}$$

and

$$c\left(x\right) = \exp\left(-\left(x/k\right)^2\right) \tag{9}$$

where k is the threshold parameter to be tuned for a particular application. A small k preserves weak edges, but at the same time may cause staircase effect easily. A large k , on the contrary, overcomes the staircase effect, but at a cost of severely degrading the edges. It is quite clear that k plays an important role in the diffusion process.
